# Frailty as a predictor of future falls and disability: a four-year follow-up study of Chinese older adults

**DOI:** 10.1186/s12877-020-01798-z

**Published:** 2020-10-06

**Authors:** Quan Zhang, Xinyi Zhao, Huiying Liu, Hua Ding

**Affiliations:** 1 School of Health Humanities, Peking University, No. 38 Xueyuan Road, Beijing, 100191 China; 2 National School of Development, Peking University, No. 5 Yiheyuan Road, Beijing, 100871 China; 3 Department of Sociology, Central South University, No. 932 Lushannan Road, Changsha, 410083 Hunan Province China; 4 Social Survey and Opinion Research Centre, Central South University, No. 932 Lushannan Road, Changsha, 410083 Hunan Province China; 5 Institute of Social Science Survey, Peking University, No. 5 Yiheyuan Road, Beijing, 100871 China

**Keywords:** Frailty phenotype, Falls, Incident disability, Worsening disability, Cohort study

## Abstract

**Background:**

Frailty, which is defined as aging-related multisystem impairments, can lead to adverse health outcomes. However, evidence for such a connection in Chinese older adults remains lacking. This study examined the association between frailty and future falls and disability among community-dwelling Chinese older adults.

**Methods:**

Data were obtained from the 2011 and 2015 waves of the China Health and Retirement Longitudinal Study. Participants were aged 60 years and above at baseline in 2011 and completed the follow-up survey in 2015. Outcome measures were future falls, incident disability in activities of daily living (ADLs) and instrumental activities of daily living (IADLs), and worsening performance of ADLs and IADLs. A multivariate logistic regression was conducted to examine the association between frailty phenotype and falls, incident disability, and worsening disability during a four-year period.

**Results:**

We found that frail participants were at increased risk at follow-up for: falls (OR 1.54, 95% CI, 1.14–2.08); developing new ADL difficulties (OR 4.10, 95% CI, 2.79–6.03) and IADL difficulties (OR 3.06, 95% CI, 2.03–4.61); and worsening ADLs performance (OR 2.27, 95% CI, 1.27–4.06), after adjusting for potential confounders. Prefrailty was also significantly associated with future falls, incident disability in ADLs and IADLs, but with a lower magnitude of effect.

**Conclusions:**

Frailty phenotype is an independent predictor of future falls, incident disability, and worsening performance in ADLs among Chinese older adults. The association suggests the need to pay special attention in caring for frail and prefrail elders and improving individuals’ frailty status.

## Background

Frailty, defined as the presence of multisystem impairment and vulnerability [1, 2], can result in decreased resilience to stressor events [3, 4], thereby increasing the risks for multiple adverse health outcomes, including falls [5], fractures [6], disability, morbidity [7, 8], and mortality [7, 9]. Frailty’s association with these adverse outcomes can bring increased care needs and increased use of hospital care and long-term care [4]. Such a challenge is likely to be more evident in China, which has the largest aging population (250 million aged 60 and older in 2018, accounting for 17.9% of the population [10]), and a high prevalence of prefrailty (51.2%) and frailty (7.0%) among people aged 60 years and above [3]. Therefore, understanding the relationship between frailty and adverse outcomes among Chinese elderly has implications for future health and social care planning.

A growing body of literature has documented a positive association between frailty and future falls [5, 6, 11, 12], but no consensus has been reached regarding the magnitude of the effect. In addition, most of those studies were conducted in developed countries, leaving the relationship insufficiently examined in developing countries such as China. To our knowledge, only limited evidence can be found for China: in Rugao [13, 14] and Beijing [15]. Cai et al. [16] used national data from the China Health and Retirement Study (CHARLS) to reveal the cross-sectional association between frailty and falls, but the prospective effects remain unknown.

The association between frailty and disability has been extensively examined and has shown that frailty is predictive of a range of disability outcomes, including incident ADL disability and IADL disability [8, 17, 18], worsening ADL disability and IADL disability [8, 19], and poor recovery from ADL disability and IADL disability [20]. However, few studies have comprehensively considered different disability outcomes at the same time, including both the incidence and deterioration of disabilities. In addition, there has only been cross-sectional evidence suggesting the association between frailty and disability among Chinese older adults [3, 21, 22].

In the present study, we used data from CHARLS, a nationally representative four-year follow-up study in China, to examine the associations between frailty and future falls, between frailty and incident ADL disability, and IADL disability among those free of disability at baseline, and between frailty and worsening ADL disability and IADL disability among those already disabled at baseline.

## Methods

### Data and participants

Data for this study originated from the 2011 and 2015 waves of the CHARLS survey, a nationally representative longitudinal survey of the middle-aged and elderly population (aged 45 and above) and their spouses in China, collecting information such as basic demographics, family information, health status, health care, employment, and household economy. The CHARLS survey derives from strict adherence to a multi-stage stratified probability proportional to size (PPS) sampling, ensuring that the baseline sample closely matched the 2010 census in demographics. A total of 17,705 respondents were interviewed at the baseline survey in 2011 and were followed every two years afterward using a face-to-face, computer-assisted personal interview (CAPI) technique (for more details, see Zhao et al. [23]).

To fulfill our purpose, we chose an initial sample of 5874 participants who were aged 60 and above at baseline in 2011 and had completed the follow-up survey in 2015. We focused on those aged 60 and above because the age of 60 is widely considered as the onset of old age among Chinese people, and the retirement age in China is 50/55 for females and 60 for males. Frailty was measured at baseline; data on falls and disability were collected at baseline and the 2015 follow-up. Specifically, in our analysis of falls, we used 4349 participants with no missing values in the frailty assessment, falls assessment at baseline and follow-up, and other covariates. For our incident ADL and IADL disability analyses, we included participants who had no disability in 2011 but did have a disability assessment in 2015, and who had no missing values in the frailty measurement and other covariates. This resulted in an analytic sample of 3382 participants for incident ADL disability analysis and 3178 for incident IADL disability analysis. Participants meeting the following criteria were used for an analysis of worsening performance of ADLs and IADLs: 1) having disability in 2011; 2) having a disability assessment in 2015; and 3) having no missing values with frailty measurement and other covariates. This led to an analytic sample of 895 for an analysis of worsening ADL performance and a sample of 1050 for worsening IADL performance.

### Frailty assessment

Frailty was assessed by using a modified version of Fried et al.’s [24] scale, the most widely used and validated physical frailty phenotype (PFP) scale, which includes five elements: weakness, slowness, exhaustion, inactivity, and shrinking. Weakness was defined as a maximum handgrip strength below or equal to the 20th percentile of the population distribution, with adjusting for sex and body mass index (BMI). In defining slowness, we relied on two repetitions of a 2.5-m walking test at normal speed in CHARLS, which was valid measurements of gait speed although 5 to 10 m length was the recommended timed distance [25]. CHARLS respondents started by lining their feet up at the starting point and walked past the other end of the tape before they stopped [23]. Slowness was defined as the slowest 20% of the population distribution, based on the average time required to walk the 2.5-m course, with adjusting for sex and height. The specific cut-points for defining weakness and slowness were referred to in previous research using the same survey [3]. Exhaustion was coded as *yes* if participants answered either “occasionally” or “all of the time” to either of two questions from the modified Centre for Epidemiological Studies-Depression (CES-D) scale [26]: “I could not get going” and “I felt everything I did was an effort.” Participants were identified as inactive if they self-reported that they did not walk continuously for 10 min or more during a typical week. Participants met our criteria for shrinking if they had a BMI of 18.5 kg/m^2^ or less, or if they reported that they had lost 5 or more kilograms in the previous year. The respondents’ frailty level was identified for those who had information relevant to at least four frailty measures. Individuals that met three to five criteria were classified as “frail,” and those who met one or two criteria were considered “prefrail.” If no frailty indicators applied, the individuals were classified as “nonfrail.”

### Outcome measures

The outcomes of falls, incident disability, and worsening disability were assessed. Falls were assessed using the following question: “Have you fallen down in the last two years?” Fall status was dichotomized into having had no falls or having had falls in the previous two years. We assessed ADL disability as having difficulty with at least one task of ADLs (dressing, bathing, eating, getting into or out of bed, toileting, and urination), and we identified IADL disability as having difficulty with at least one task of IADLs (doing household chores, cooking, shopping, managing money, taking medicine). We defined incident disability as developing any new difficulty in ADLs or IADLs by 2015, and we computed it only for participants who had been free of disability at baseline. Worsening disability was defined by an increase in the number of reported difficulties in performing ADLs or IADLs in 2015 with respect to baseline, among subjects who were already disabled in their ADLs or IADLs in 2011.

### Control variables

Sociodemographic characteristics included age (in years), sex, marital status (married vs. unmarried), urban residence (urban vs. rural), educational level (below primary school education vs. primary school education and above), and pension status. Baseline morbidity was considered as a potential confounder. CHARLS respondents were required to answer whether they had been diagnosed to have several chronic conditions. In all regression analyses, self-reported doctor diagnosis or self-knowledge of diabetes, heart problems, arthritis, stroke, memory-related diseases, emotional problems, nervous issues, or psychiatric problems, cancer, and hypertension were included as control variables. We also adjusted for baseline smoking, drinking, and BMI. We categorized BMI into three groups: underweight (BMI ≤ 18.5); overweight (BMI ≥ 25), and normal BMI (18.5 < BMI < 25).

### Statistical analyses

Baseline characteristics were described for participants in our analysis of falls, incident ADL disability analysis, incident IADL disability analysis, worsening of ADL performance analysis, and worsening of IADL performance analysis.

We constructed two multivariate models to analyse the independent effect that frailty phenotype at baseline exerted on falls over a four-year period. In the minimally adjusted model, we adjusted only for the demographic variables of age, sex, marital status, and falls at baseline. In the fully adjusted model, we further adjusted for socioeconomic status, including urban residence, educational level, and pension status, and also for baseline self-reported heart problems, arthritis, stroke, memory-related disease, emotional problems, and cancer. Two multivariate models were computed for each disability outcome (incident ADL disability, incident IADL disability, worsening of ADL performance, and worsening of IADL performance). The first model adjusted for age, sex, and marital status; the second model further adjusted for SES, baseline self-reported heart problems, arthritis, stroke, memory-related disease, emotional problems, hypertension, smoking, drinking, BMI, and falls in 2011.

Multivariate odds ratios (ORs) from the logistical model were reported, along with the 95% confidence interval (CI), and asterisks indicate the significance levels of *p*-values. All analyses were conducted using the statistical software Stata 14.

## Results

### Characteristics of the study participants

Table 1 shows the baseline characteristics of the participants included in our analyses of falls, incident disability in ADLs and in IADLs, and worsening disability in ADLs and IADLs. Of the 4349 participants in the falls analysis, the mean age was 67.1 years with a median of 66, 75th percentile of 71, and 90th percentile of 76 years, 49.2% were female, and 36.0% were nonfrail, 57.2% were prefrail, and 6.8% were frail. The distribution of demographic characteristics was similar among the participants included in the analyses of incident ADL disability and IADL disability. However, participants in the worsening ADL disability and IADL disability analyses had a very different demographic distribution, with higher mean ages (68.0 and 68.4, respectively), and substantially larger proportions of females (56.5 and 60.5%, respectively). The frail proportion of the participants in the worsening disability analysis (for participants who were already disabled at baseline) was approximately three times higher than that among participants in the incident disability analysis (participants who were free of disability at baseline).

**Table 1:** Characteristic of study participants

		Fall analysis group	Incident ADL disability group	Incident IADL disability group	Worsening of ADL disability group	Worsening of IADL disability group
Characteristics	*N*	4349	3382	3178	895	1050
Frailty phenotype	Non-frail	1564 (36.0)	1398 (41.3)	1325 (41.7)	140 (15.6)	210 (20.0)
	Prefrail	2489 (57.2)	1826 (54.0)	1718 (54.1)	622 (69.5)	692 (65.9)
	Frail	296 (6.8)	158 (4.7)	135 (4.2)	133 (14.9)	148 (14.1)
Fall 2009 to 2011, No (%)	No	3525 (81.1)	2853 (84.4)	2669 (84.0)	612 (68.4)	753 (71.7)
	Yes	824 (18.9)	529 (15.6)	509 (16.0)	283 (31.6)	297 (28.3)
Age, mean (SD)	Age	67.1 (6.0)	66.9 (6.0)	66.6 (5.7)	68.0 (6.2)	68.4 (6.8)
Sex, No (%)	Male	2209 (50.8)	1782 (52.7)	1731 (54.5)	389 (43.5)	415 (39.5)
	Female	2140 (49.2)	1600 (47.3)	1447 (45.5)	506 (56.5)	635 (60.5)
Marital Status, No (%)	Unmarried	864 (19.9)	644 (19.0)	573 (18.0)	208 (23.2)	267 (25.4)
	Married	3485 (80.1)	2738 (81.0)	2605 (82.0)	687 (76.8)	783 (74.6)
Residence, No (%)	Rural	2898 (66.6)	2175 (64.3)	2034 (64.0)	673 (75.2)	777 (74.0)
	Urban	1451 (33.4)	1207 (35.7)	1144 (36.0)	222 (24.8)	273 (26.0)
At least primary education, No (%)	No	2467 (56.7)	1818 (53.8)	1646 (51.8)	599 (66.9)	731 (69.6)
	Yes	1882 (43.3)	1564 (46.2)	1532 (48.2)	296 (33.1)	319 (30.4)
Any pension, No (%)	No	2288 (52.6)	1675 (49.5)	1566 (49.3)	582 (65.0)	662 (63.0)
	Yes	2061 (47.4)	1707 (50.5)	1612 (50.7)	313 (35.0)	388 (37.0)
Diabetes, No (%)	No	4082 (93.9)	3186 (94.2)	2997 (94.3)	827 (92.4)	967 (92.1)
	Yes	267 (6.1)	196 (5.8)	181 (5.7)	68 (7.6)	83 (7.9)
Heart problems, No (%)	No	3711 (85.3)	2921 (86.4)	2748 (86.5)	724 (80.9)	857 (81.6)
	Yes	638 (14.7)	461 (13.6)	430 (13.5)	171 (19.1)	193 (18.4)
Arthritis, No (%)	No	2685 (61.7)	2273 (67.2)	2112 (66.5)	374 (41.8)	504 (48.0)
	Yes	1664 (38.3)	1109 (32.8)	1066 (33.5)	521 (58.2)	546 (52.0)
Stroke, No (%)	No	4231 (97.3)	3319 (98.1)	3121 (98.2)	842 (94.1)	992 (94.5)
	Yes	118 (2.7)	63 (1.9)	57 (1.8)	53 (5.9)	58 (5.5)
Memory-related disease, No (%)	No	4263 (98.0)	3336 (98.6)	3137 (98.7)	858 (95.9)	1011 (96.3)
	Yes	86 (2.0)	46 (1.4)	41 (1.3)	37 (4.1)	39 (3.7)
Emotional problems, No (%)	No	4255 (97.8)	3331 (98.5)	3139 (98.8)	854 (95.4)	997 (95.0)
	Yes	94 (2.2)	51 (1.5)	39 (1.2)	41 (4.6)	53 (5.0)
Cancer, No (%)	No	4318 (99.3)	3353 (99.3)	3153 (99.4)	883 (99.0)	1036 (99.1)
	Yes	31 (0.7)	22 (0.7)	20 (0.6)	9 (1.0)	9 (0.9)
Hypertension, No (%)	No	3029 (69.8)	2411 (71.3)	2265 (71.3)	577 (64.5)	691 (65.8)
	Yes	1308 (30.2)	971 (28.7)	913 (28.7)	318 (35.5)	359 (34.2)
Smoker, No (%)	No	2972 (68.3)	2271 (67.1)	2117 (66.6)	658 (73.5)	784 (74.7)
	Yes	1377 (31.7)	1111 (32.9)	1061 (33.4)	237 (26.5)	266 (25.3)
Drinker, No (%)	No	2990 (68.8)	2295 (67.9)	2128 (67.0)	648 (72.4)	787 (75.0)
	Yes	1359 (31.2)	1087 (32.1)	1050 (33.0)	247 (27.6)	263 (25.0)
BMI, No (%)	Normal	2779 (64.2)	2208 (65.3)	2044 (64.3)	539 (60.2)	669 (63.7)
	Underweight	425 (9.8)	326 (9.6)	291 (9.2)	93 (10.4)	120 (11.4)
	Overweight	1124 (26.0)	848 (25.1)	843 (26.5)	263 (29.4)	261 (24.9)

### Frailty and falls

Table 2 shows that frailty was an independent predictor of falls at follow-up. Of the 4349 participants in our analysis of falls, 824 (19.0%) had fallen at least once between 2013 and 2015. The rate of falls ranged from 12.1% for nonfrail participants to 27.4% for frail participants. When age, sex, marital status, and falls at baseline had been adjusted for, the odds of falls were 35% higher among prefrail individuals (*p* < 0.001) and 81% higher among frail individuals (*p* < 0.001) than they were for the nonfrail. In the fully adjusted model, prefrail participants were more likely to fall, with an OR of 1.24 (*p* < 0.05), and frail participants had an even greater likelihood, at an OR of 1.54 (*p* < 0.01).

**Table 2:** Impact of frailty status on experience of fall

Frailty status	Fall No. (%)	Minimally adjusted^a^		Fully adjusted^b^	
		OR	95% CI	OR	95% CI
*N* = 4349	824 (19.0)				
Non-frail	189 (12.1)	Ref.		Ref.	
Prefrail	554 (22.3)	1.35^***^	[1.15,1.60]	1.24^*^	[1.05,1.47]
Frail	81 (27.4)	1.81^***^	[1.35,2.43]	1.54^**^	[1.14,2.08]

Note: a. Age, sex, marital status, and fall in 2011 were adjusted

b. Urban residence, education, any pension, and self-report diagnosis of diabetes, heart problems, arthritis, stroke, memory-related disease, emotional, nervous, or psychiatric problems, and cancer in 2011 were further adjusted

^*^ *p* < 0.05, ^**^ *p* < 0.01, ^***^ *p* < 0.001

### Frailty and incident ADL disability or IADL disability

Table 3 shows the probabilities of incident ADL disability and incident IADL disability among participants with different frailty levels. The chance of incident ADL disability increased with frailty levels: 15.0% of the nonfrail, 28.0% of the prefrail, and 46.2% of the frail had developed incident disability in ADLs by 2015. In the minimally adjusted model, being prefrail was associated with a 2.06-times greater likelihood of incident disability in ADLs (*p* < 0.001) than being nonfrail was. The difference between nonfrail and frail was even more pronounced, with the frail respondents having a more than four-fold risk of developing ADL difficulties (*p* < 0.001). Additionally, controlling for socioeconomic status (SES), self-reported diagnosis of disease, smoking, drinking, and BMI did not weaken the association between frailty and incident ADL disability, and it even increased the odds among frail participants.

**Table 3:** Impact of frailty status on incident disability

Incident disability	Frailty status	Incidence No. (%)	Minimally adjusted^a^		Fully adjusted^b^	
			OR	95% CI	OR	95% CI
ADL	*N* = 3382	794 (23.5)				
disability	Non-frail	210 (15.0)	Ref.		Ref.	
	Prefrail	511 (28.0)	2.06^***^	[1.72,2.47]	2.02^***^	[1.68,2.44]
	Frail	73 (46.2)	4.01^***^	[2.81,5.72]	4.10^***^	[2.79,6.03]
IADL	*N* = 3178	816 (25.7)				
disability	Non-frail	216 (16.3)	Ref.		Ref.	
	Prefrail	540 (31.4)	2.22^***^	[1.86,2.66]	2.04^***^	[1.69,2.46]
	Frail	60 (44.4)	3.48^***^	[2.39,5.08]	3.06^***^	[2.03,4.61]

Note: a. Age, sex, and marital status were adjusted

b. Urban residence, education, any pension, smoke, drink, self-report diagnosis of diabetes, heart problems, arthritis, stroke, memory-related disease, emotional, nervous, or psychiatric problems, and hypertension, BMI and fall in 2011 were further adjusted

^*^ *p* < 0.05, ^**^ *p* < 0.01, ^***^ *p* < 0.001

Frailty level was also shown to be a statistically significant predictor of incident acquisition of an IADL disability, even after fully adjusting for potential confounders, but with a smaller magnitude of effect than its prediction strength for an incident ADL disability (Table 3). After adjusting for age, sex, and marital status, being either prefrail or frail was associated with increased odds of acquiring an incident IADL disability (OR = 2.22 and 3.48, *p* < 0.001) compared with the odds for the nonfrail. The strength of association between frailty and acquiring an incident IADL disability was slightly attenuated but still significant (*p* < 0.001) in the fully adjusted model, for both prefrail (OR = 2.04) and frail (OR = 3.06) participants.

### Frailty and worsening of an ADL or IADL disability

Table 4 shows that among 895 individuals that were already ADL disabled at baseline, 210 (23.5%) experienced an increase in the number of their ADL difficulties by 2015. The probability of worsening ADL performance was higher among the prefrail (21.5%) and frail persons (37.6%) than it was among those that were nonfrail (18.6%). The odds of a worsening ADL performance were 19% (minimally adjusted) higher and 3% (fully adjusted) higher among prefrail persons than the odds were for the nonfrail participants, but the association did not reach statistical significance. The likelihood of experiencing a worsening ADL performance was significantly higher among frail individuals than it was among nonfrail individuals in both the minimally adjusted model (OR = 2.52, *p* < 0.01) and the fully adjusted model (OR = 2.27, *p* < 0.01).

**Table 4:** Impact of frailty status on worsening disability

Worsening	Frailty status	Worsening No. (%)	Minimally adjusted^a^		Fully adjusted^b^	
disability			OR	95% CI	OR	95% CI
ADL	*N* = 895	210 (23.5)				
difficulties	Non-frail	26 (18.6)	Ref.		Ref.	
	Prefrail	134 (21.5)	1.19	[0.75,1.91]	1.03	[0.63,1.67]
	Frail	50 (37.6)	2.52^**^	[1.44,4.40]	2.27^**^	[1.27,4.06]
IADL	*N* = 1050	206 (19.6)				
difficulties	Non-frail	32 (15.2)	Ref.		Ref.	
	Prefrail	133 (19.2)	1.24	[0.81,1.89]	1.21	[0.78,1.88]
	Frail	41 (27.7)	1.70	[0.99,2.92]	1.63	[0.93,2.86]

Note: a. Age, sex, and marital status were adjusted

b. Urban residence, education, any pension, smoke, drink, self-report diagnosis of diabetes, heart problems, arthritis, stroke, memory-related disease, emotional, nervous, or psychiatric problems, and hypertension, BMI and fall in 2011 were further adjusted

^*^ *p* < 0.05, ^**^ *p* < 0.01, ^***^ *p* < 0.001

Experiencing a worsening IADL performance by 2015 was observed for 206 (19.6%) of the 1050 participants that were already IADL disabled at baseline. The proportion that had an increased number of IADL difficulties declined steadily from the group of those who were frail to the group of those who were nonfrail. More than one-fourth of the frail had a decline in their IADL performance by 2015, whereas only 15.2% of the nonfrail did. The fully adjusted model did not show a statistically significant association between frailty level and a decline in IADL performance.

## Discussion

Using a nationally representative four-year follow-up study in China, we found that the frailty phenotype was significantly associated with a range of adverse outcomes, including falls, incident disability in ADLs/IADLs, and worsening ADL disability among individuals aged 60 and older. The findings indicated that frailty was a predictor of future falls, even after adjusting for sociodemographic characteristics, baseline morbidity, and falls. Frailty was shown to be significantly associated with a newly developed disability in ADLs and/or IADLs in 2015 among participants who had been free of disability at baseline. In addition, among the participants who were disabled in ADLs at baseline, the risk of functional decline was significantly higher among frail individuals than it was among nonfrail elders.

The results presented here are in line with previous studies showing frailty to be a predictor of future falls [5, 6, 11, 12]. For instance, using data from the Global Longitudinal Study of Osteoporosis in Women (GLOW), Tom et al. [6] found that the frailty phenotype was associated with a greater risk of falls among females aged 55 years and above in Australia, Europe, and North America. The association between frailty and future falls also occurred in this study when psychological and cognitive markers were incorporated, which again was in line with the results from previous studies [11]. A greater risk of falling among frail elder people is likely to be attributable to a decrease in their functional ability to maintain balance and an increase in their vulnerability to accidents or disease symptoms. Besides, frailty phenotype contains components like slowness, weakness, exhaustion, etc., which have been found to increase the risk of falls [27, 28]. Whereas the strength of such associations in our study coincides with previous studies, the magnitude of the predictive ability differs from study to study. The fully adjusted ORs of prefrailty and frailty in our study were quite similar to, but slightly lower than, the pooled ORs from a systemic review focusing on community-dwelling older people [5]. Our lower OR findings may be the result of having used a different population regarding their baseline characteristics and different lengths of follow-up.

Our finding that baseline frailty was strongly associated with incident disability and worsening disability coincides with results from previous studies [6, 8, 18, 19]. For example, using the Survey of Health, Aging and Retirement in Europe (SHARE), Macklai et al. [8] showed that at a two-year follow up, frail individuals were at increased risk for developing ADL and IADL difficulties and experiencing an increase in the number of ADL difficulties. Additional solid evidence was provided in two systematic reviews showing that frail people were more likely to have incident disability [18, 19] and worsening disability [19]. The present study extended previous research by using national representative data from China, which has the largest population of elderly people in the world. The association between frailty and disability might be explained by the links that frailty has with increased inflammation, elevated markers of blood clotting [29], atherosclerosis [30], and chronic renal insufficiency [31], all of which can contribute to the acceleration of chronic disease and disability. Interestingly, the association between prefrailty and increasing ADL disability did not reach statistical significance in this study. A plausible explanation for that lack of association could be that prefrailty is defined as the presentation of only one or two frailty components, while difficulty in performance of ADLs such as toileting and eating is a very serious condition, thus prefrailty is not likely to pose such a severe impact on older adults’ functional health. Furthermore, in contrast to the findings of previous studies [8], we found that the association between frailty and worsening IADL performance was insignificant in the fully adjusted model. That discrepancy could be attributed to the respondents in the present study were relatively younger, as the samples in most of the previous studies documenting a significant positive association between frailty and worsening ADL/IADL had a higher mean age (higher than 70 year-old) [2, 8]. Some other possible causes may be our relatively smaller sample size after restricting our sample to those already disabled at baseline (1050 vs. 10,133 participants), worsening IADL performance over a longer period (4 years vs. 2 years), and our additional adjustments for smoking, drinking, BMI, memory-related diseases, and emotional problems beyond sociodemographic characteristics and baseline morbidity.

Several limitations must be noted when interpreting our findings. First, frailty was assessed only once, at baseline, before we conducted the follow-up assessments of falls, incident disability, and worsening disability. Therefore, we could not rule out the possibility of transitions in frailty status during the follow-up. Second, our measures of falls and disabilities relied exclusively on self-reported information. The accuracy of such information may have been compromised due to memory disorders, especially among older people, leaving open the possibility that the results could differ if more objective measures were used. Third, selection bias might exist regarding our longitudinal sample, given that individuals who did not complete the household survey and physical test at both two waves were dropped. The participants who remained in our analyses might be younger and healthier than those who were excluded. Cautious consideration should be taken when generalizing the findings of this study.

## Conclusions

In summary, we examined the association between elderly people’s frailty phenotype and their likelihood of future falls, incident disability, and worsening disability. Our results provided additional evidence that frailty is a strong and independent predictor of falls, newly developed difficulties with ADLs and IADLs, and declines in function related to several ADL difficulties among Chinese older adults. In light of the increased likelihood of adverse outcomes with increasing frailty level, special attention should be paid to the care of frail and prefrail elders. In addition, in an effort to prevent the occurrence of adverse outcomes, effective measures such as physical exercise and nutritional intervention should be undertaken to improve elder individuals’ frailty status and slow the progression of their frailty.

## Data Availability

The raw data is available on the website (http://charls.pku.edu.cn/en).

## Abbreviations

ADL: Activities of daily living
IADL: Instrumental activities of daily living
OR: Odds ratio
CI: Confidence interval
CHARLS: China Health and Retirement Longitudinal Study
PFP: Physical frailty phenotype
BMI: Body mass index
CES-D: Center for epidemiologic studies - depression Scale
